# Association of damage to the coracohumeral ligament with anterosuperior rotator cuff degeneration revealed by anatomical dissection

**DOI:** 10.1038/s41598-022-08070-x

**Published:** 2022-03-10

**Authors:** Lukas Unerfußer, Gilbert Manuel Schwarz, Lena Hirtler

**Affiliations:** 1 Division of Anatomy, Center for Anatomy and Cell Biology, Medical University of Vienna, Währinger Straße 13, 1090 Vienna, Austria; 2 Division of Traumatology, Department of Orthopedic and Trauma Surgery, Medical University of Vienna, Währinger Gürtel 18-20, 1090 Vienna, Austria

**Keywords:** Musculoskeletal system, Ligaments, Muscle, Tendons

## Abstract

The coracohumeral ligament (CHL) is an important structure of the biceps pulley which also merges with the rotator cuff. Which role it actually plays in the pathogenesis of rotator cuff degeneration (RCD) and rotator cuff tears (RCT) is still a point of discussion. The hypothesis of this study was, that macroscopic injury to the anterosuperior part of the rotator cuff also includes parts of or the whole CHL. Forty fresh-frozen shoulders were dissected and examined, the morphology of the rotator cuff and the coracohumeral ligament were evaluated and existing lesions documented. 27.5% of the shoulder joints showed an anterosuperior full-thickness RCT. 57.5% of all examined shoulder girdles showed at least a partial rupture of the CHL. A highly significant correlation (*p* < 0.001, rho = 0.529) between the presence of rotator cuff tears and ruptures of the CHL was found. Cartilage damage within the anterosuperior section of the humeral head was observed in 20% cases. In rotator cuff degeneration and atraumatic rotator cuff tears of the elderly population, the pathomechanism of full-thickness RCT is based on repetitive anterosuperior glenoid impingement. This is especially supported by the identification of a higher frequency of CHL lesions compared to RCT reported in this study. No intact CHL was identified in shoulders with damaged rotator cuff tendons.

## Introduction

Rotator cuff degeneration (RCD) and rotator cuff tears (RCT) are among the most common causes for pain and dysfunction in the shoulder girdle, their prevalence reported in literature varies overall from 5 to 39%^1–7^. This relatively large variance may occur because of different survey methods, eg. MRI, ultrasound, cadaveric dissection, and of dissimilarities in the study population, as there is a broad consensus that the prevalence of full-thickness RCT increases with age^7–9^.

Looking at the morphological distribution of tears in the rotator cuff, the supraspinatus (SSP) muscle is most often afflicted (84%), followed by the subscapularis (SSC) muscle (78%) and the infraspinatus muscle (39%)^10^. The involvement of supraspinatus and subscapularis muscles reflects the majority of anterosuperior locations in the pathogenesis of RCT, which thus also includes the so-called rotator interval.

The rotator interval, described as an opening in the tendinous rotator cuff, is bordered anteriorly by the superior border of the SSC and posteriorly by the anterior border of the SSP. It is closed and strengthened by the coracohumeral ligament (CHL), underneath pass the superior glenohumeral ligament (SGHL) and the long head of the biceps brachialis muscle (LHB)^11–13^. Thus, not only the muscles of the rotator cuff but also the complex comprised of the CHL and the SGHL are important for the stability of the shoulder joint. Especially the CHL, as it merges laterally with the tendinous part of the rotator cuff, is therefore often included in textbooks in the description of the rotator cuff^14,15^.

The CHL rises proximally from the lateral base of the coracoid process and finds its distal insertions on the lesser and greater tubercles of the humerus. Through these two osseous insertions, the CHL is subdivided into a posterolateral (plCHL) and an anteromedial (amCHL) band. Additionally, fibers of the CHL merge with the SGHL, the SSP and the SSC. It limits mostly external rotation, adduction and the descent of the humeral head and is, together with the SGHL, important to the alignment of the biceps tendon towards it entrance into the intertubercular sulcus^12,16–19^. The CHL is thus an important part of the so-called biceps pulley, which is—apart from the CHL—additionally comprised of the SGHL, the SSP and the SSC tendons^20–22^.

Due to the histological similarities of the CHL and the fibrous joint capsule (collagen-type-III) of the glenohumeral joint, the ligament is often described as a thickened part of the joint capsule^23–28^. In contrast, however, a more recent publication considers the CHL as a remnant of the tendon of the pectoralis minor muscle, which also would explain the different fiber-composition of this ligament compared to others^29^. The development of the CHL as a distinct structure is also supported by its separate embryological formation from the SGHL, the complex of both ligaments providing for the biceps pully establishing itself much later^30^.

Which role the CHL actually plays in the pathogenesis of RCT and whether it should therefore be included in the diagnosis and treatment protocols of such degenerative changes and tears is still a point of discussion^26,31^. Based on the reported location of RCT, the hypothesis of this study was, that a macroscopic visible injury to the anterosuperior part of the rotator cuff also includes parts of or the whole CHL. We therefore evaluated the morphology of the rotator cuff and the CHL through dissection in an anatomical setting.

## Results

**Figure 1:**
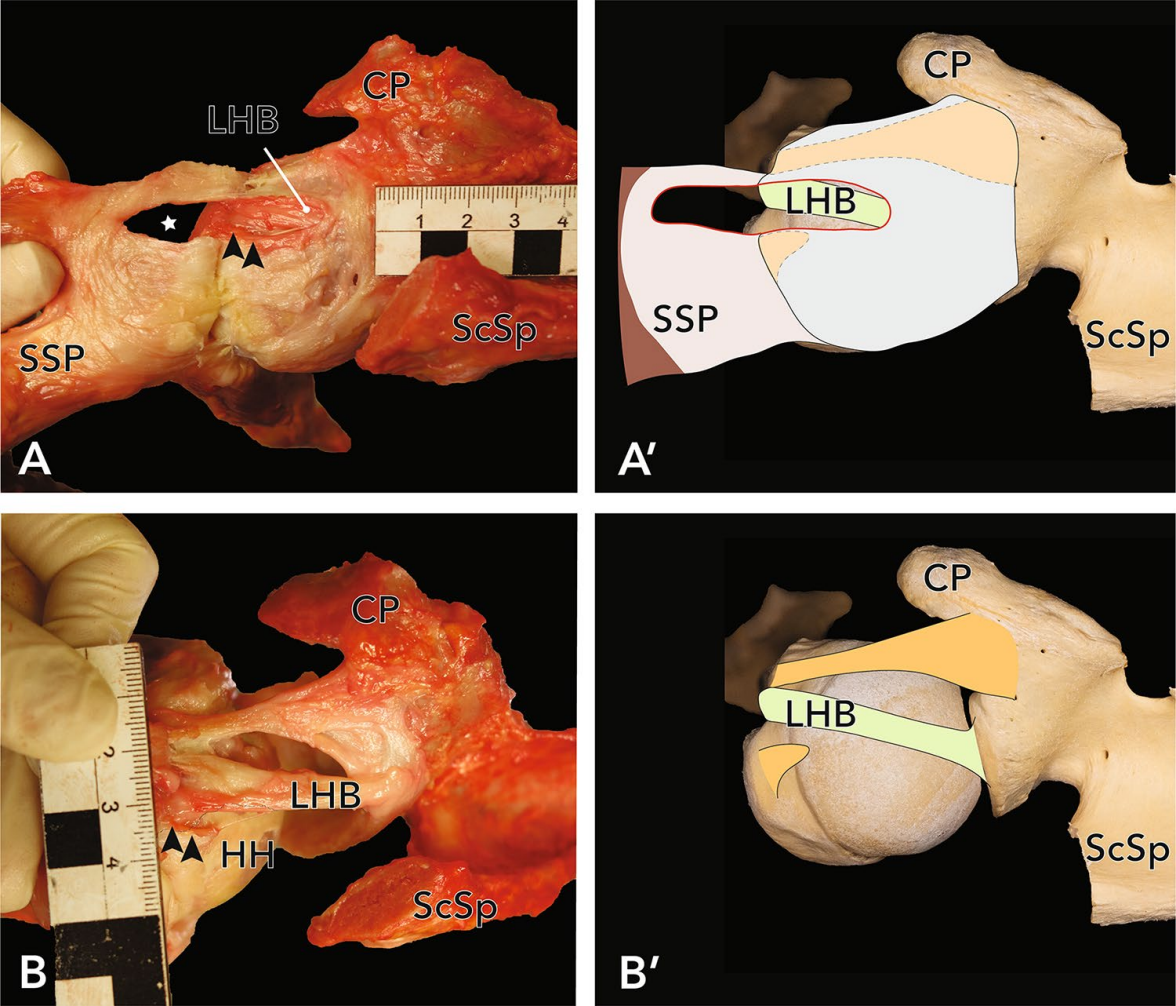
(**A**) Exemplary full-thickness SSP-lesion in a left shoulder, view from above. Asterix marks the full-thickness tear, the SSP is mobilized towards lateral. The LHB was visible through the tear. (**B**) After removal of the fibrous capsule and careful dissection of the CHL, a rupture of the posterolateral band was identified. Arrowheads mark the remnants of the posterolateral band (**B**) and its location before further dissection (**A**) for better orientation. (**A′**) and (**B′**) Approximation of the photographs as an overlay on humerus and scapula. *HH* humeral head, *CP* coracoid process, *ScSp* scapular spine, *SSP* supraspinatus muscle, *SSC* subscapularis muscle, *LHB* long head of biceps brachii muscle.

**Figure 2:**
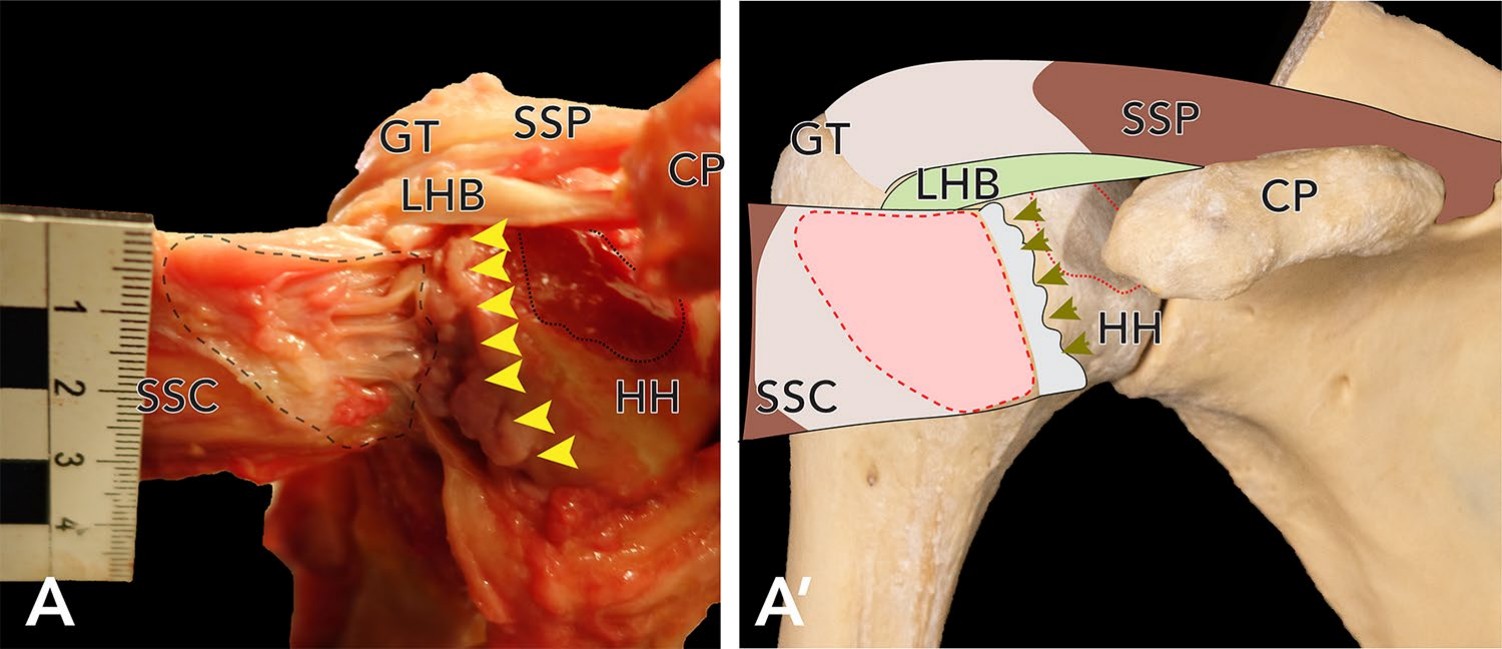
(**A**) Large partial SSC-lesion in a right shoulder, view from anterior. In this shoulder, also a large cartilage-defect was identified. Dashed line delineates the size of the SSC-lesion, dotted line delineates the cartilage-defect. Arrowheads demarcate the degenerated joint capsule. (**A′**) Approximation of the photographs as an overlay on humerus and scapula. *HH* humeral head, *CP* coracoid process, *SSC* remainder of the supscapularis muscle.

**Figure 3:**
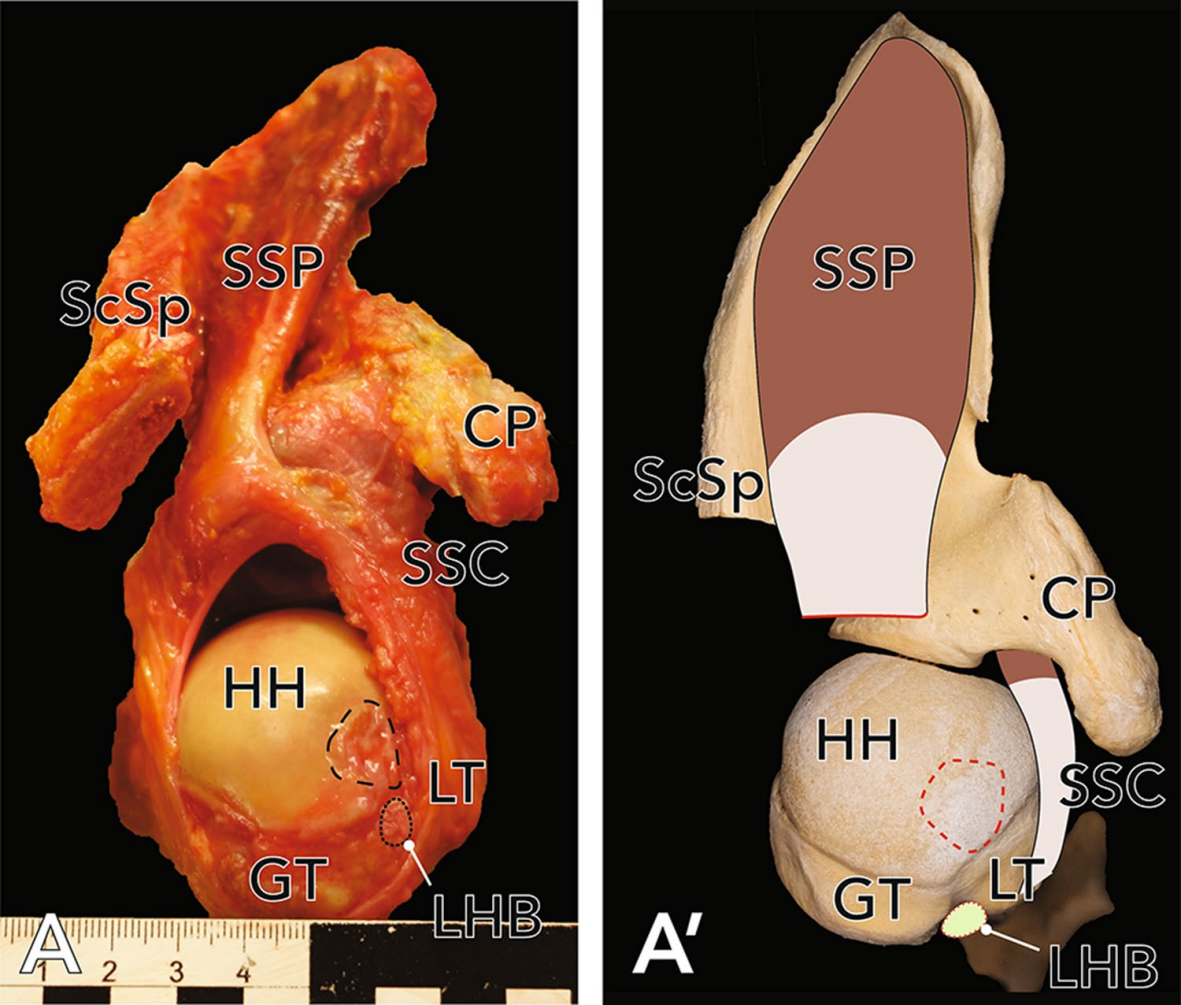
(**A**) Exemplary full-thickness SSP-lesion in a right shoulder, view from superior. The lesion was classified as Bateman III. In addition to the tendon tear, also a complete rupture of the CHL and of the LHB was identified. (**A′**) Approximation of the photographs as an overlay on humerus and scapula. Dashed line delineates an anterosuperior cartilage defect. Dotted line identifies the ruptured tendon of the LHB. *HH* humeral head, *CP* coracoid process, *ScSp* scapular spine, *GT* greater tubercle, *LT* lesser tubercle, *SSP* supraspinatus muscle, *SSC* subscapularis muscle, *LHB* long head of the biceps brachii muscle.

In 40 shoulder specimens, 27.5% (n = 11, 5 female, 6 male, 5 left, 6 right) of the shoulder joints showed an anterosuperior full-thickness rupture of the rotator cuff (close to its insertion at the greater tubercle of the humerus). In all cases of full thickness lesions the SSP tendon was affected (see Fig. 1). In addition, 3 articular-sided partial ruptures of the SSC (8%) were found (see Fig. 2). Statistical analysis revealed a mean, measured rupture area of 62.3 ± 38.4 mm^2^ (n = 9, two massive rotator cuff tears couldn’t be measured as they completely affected the tendons of SSP and SSC, see Fig. 2). There was no significant difference in sex (*p* = 0.336) and side (*p* = 0.431) in terms of the individual expanse of rotator cuff tears. The presence of RCT showed a strong positive correlation with age (*p* = 0.027, φ = 0.883). Regarding the Bateman-classification, 54.5% (n = 6) of the shoulders showed rotator cuff tears smaller than 1 cm (Bateman I tears), 36.4% (n = 4) were classified as Bateman II tears, 9.1% (n = 1) of the joints had a Bateman III lesion and two specimens (n = 2, see Fig. 3) showed a rupture of the rotator cuff wider than 5 cm (Bateman IV).

**Figure 4:**
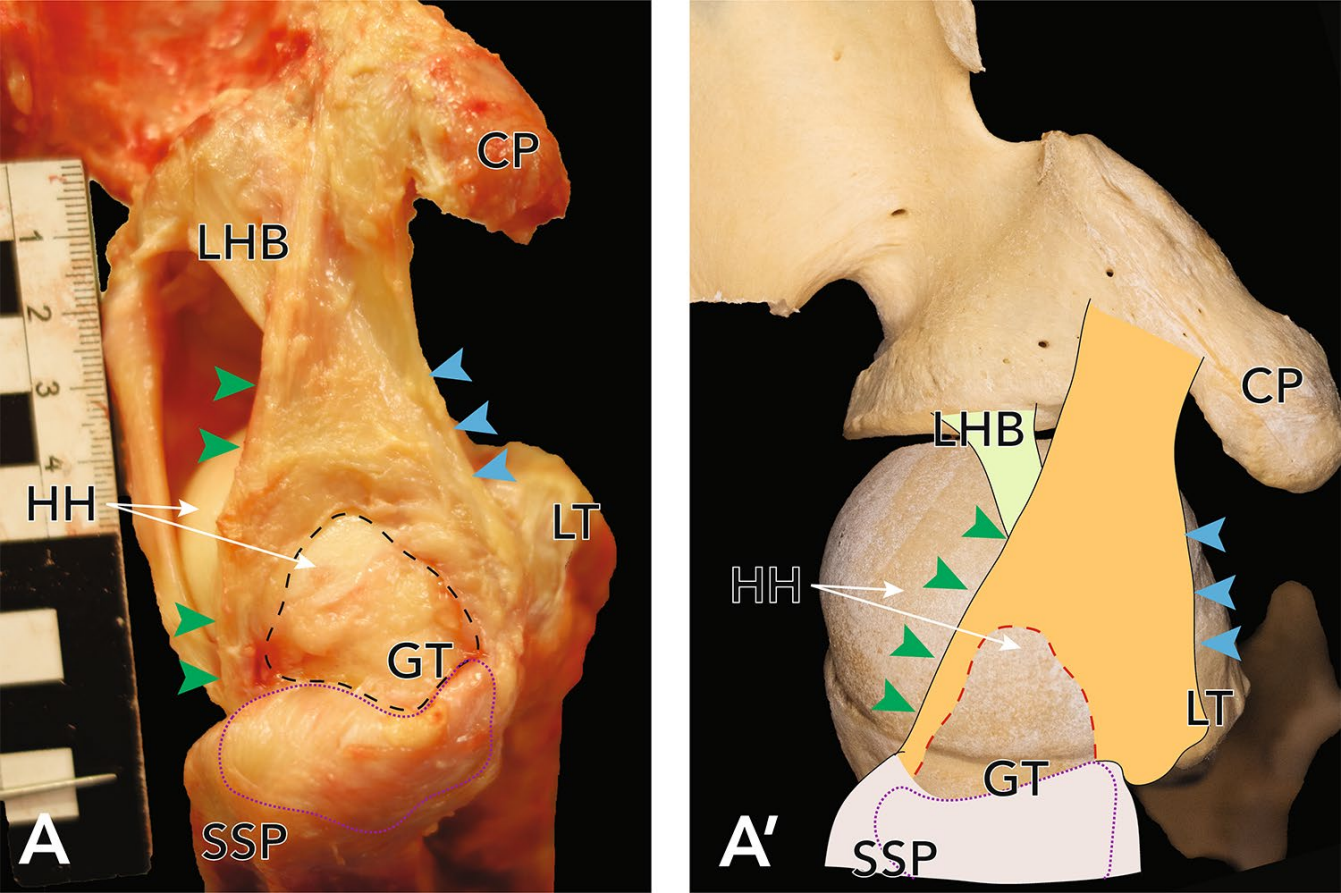
(**A**) Exemplary lesion to the posterolateral band of the CHL in a right shoulder, view from superior. (**A′**) Approximation of the photographs as an overlay on humerus and scapula. Dashed line delineates the size of the lesion, dotted line delineates the correlating partial tear of the SSP. *HH* humeral head, *CP* coracoid process, *GT* greater tubercle, *LT* lesser tubercle, *SSP* supraspinatus muscle, *LHB* long head of biceps brachii muscle.

**Figure 5:**
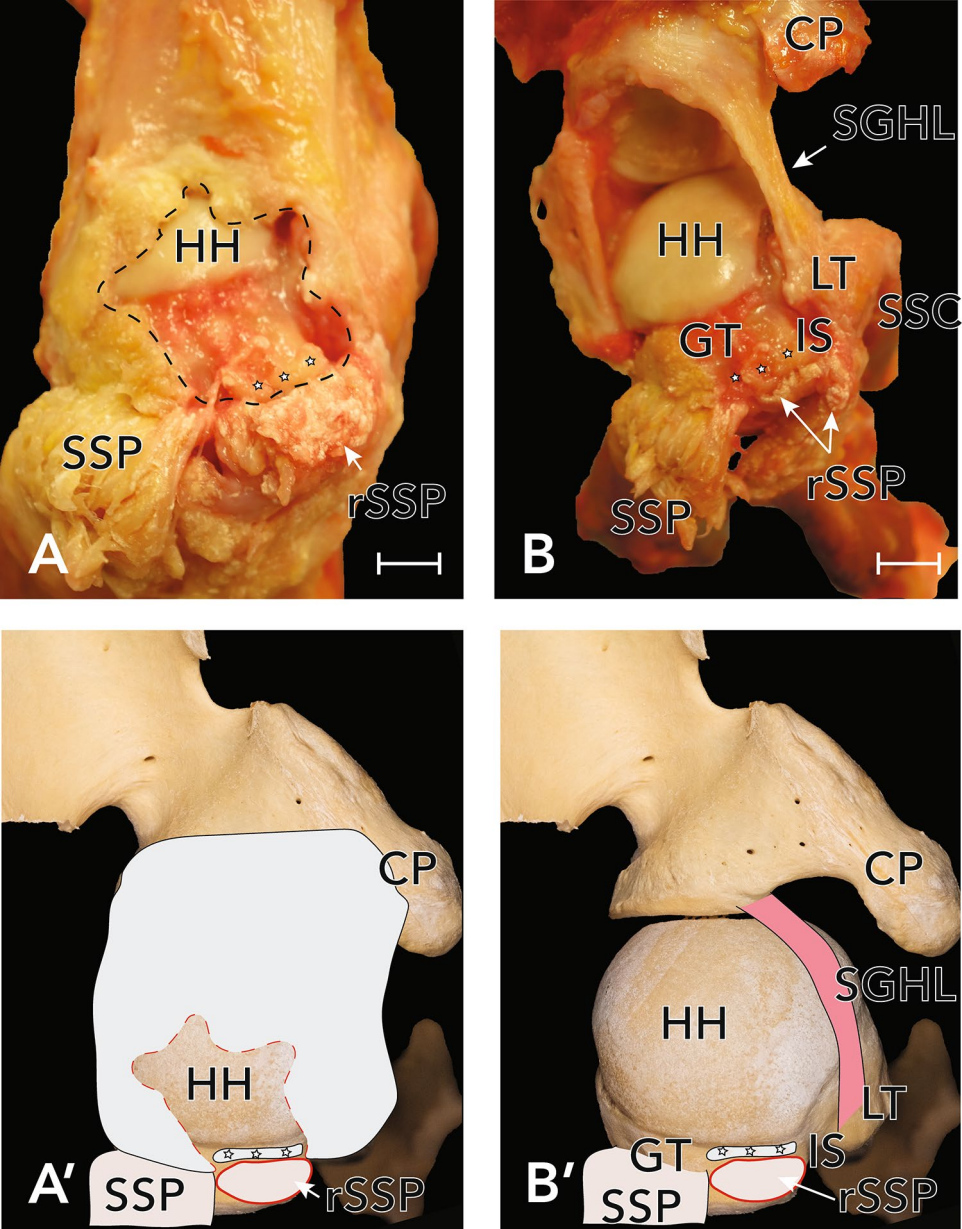
Exemplary view of a right shoulder with partial full-thickness tear of the supraspinatus tendon. (**A**) As the supraspinatus muscle was reflected, the extent of the defect in the underlying capsuloligamentous layer was observed. (**B**) A complete rupture of the CHL was identified, only the SGHL was left intact. Also, the LHB was ruptured. (**A′**) and (**B′**) Approximation of the photographs as an overlay on humerus and scapula. Asterix mark remnants of the fibrous capsule. *HH* humeral head, *CP* coracoid process, *GT* greater tubercle, *LT* lesser tubercle, *IS* intertubercular sulcus, *SGHL* superior glenohumeral ligament, *SSP* supraspinatus muscle, *SSC* subscapularis muscle.

**Figure 6:**
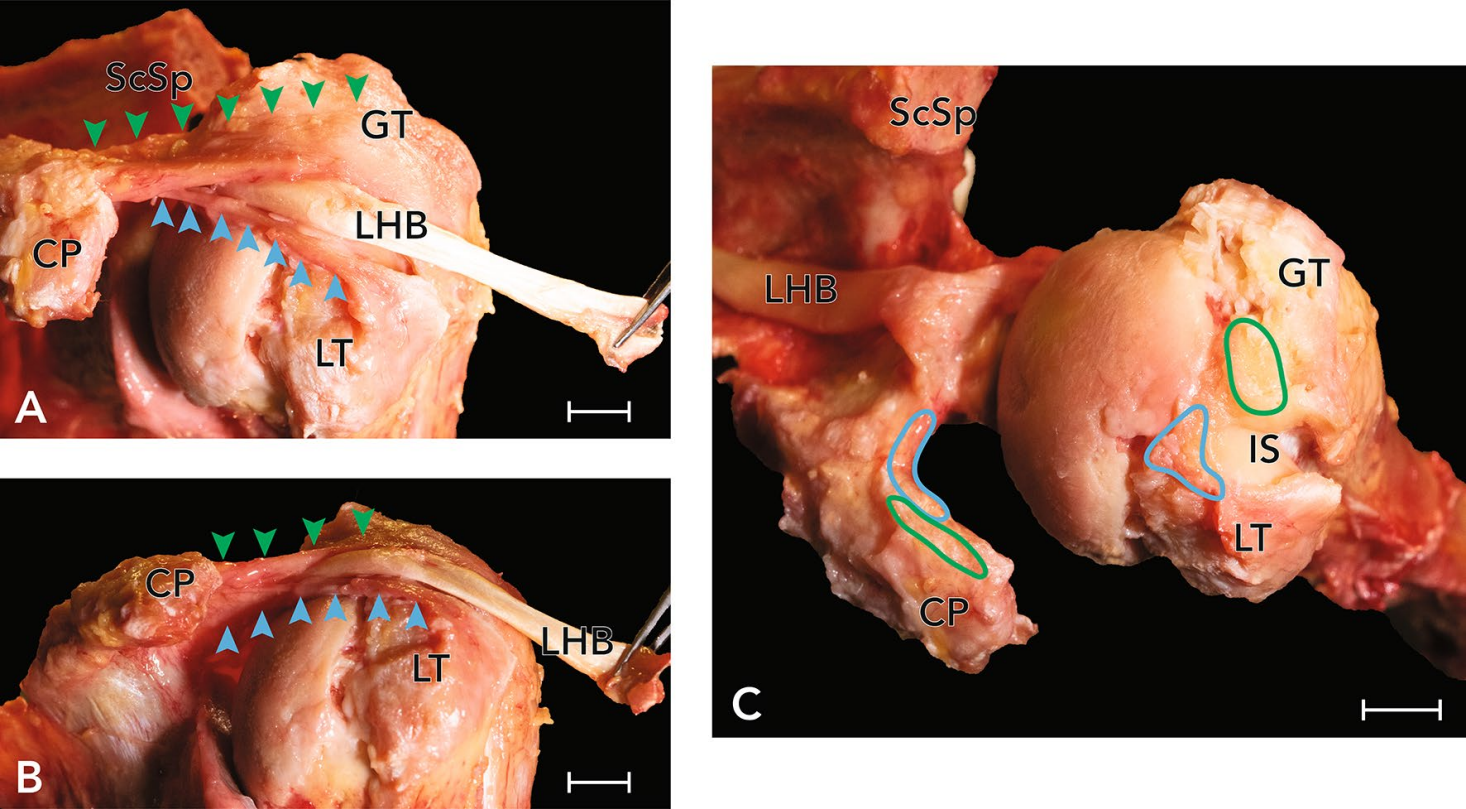
Exemplary view of the morphology of the CHL in a right shoulder. (**A**) view from anterosuperior, (**B**) view from anterior. The two bands—anteromedial (blue arrowheads) and posterolateral (green arrowheads) attached to the lesser and greater tubercle, respectively, are clearly distinguished. The LHB (extracted from the intertubercular sulcus) courses between those bands from its origin at the supraglenoidal tubercle towards the intratubercular sulcus. (**C**) The osseous attachments at the coracoid process and the humerus were marked in the photograph after removal—anteromedial band with blue markings, posterolateral band with green markings. *CP* coracoid process, *ScSp* scapular spine, *GT* greater tubercle, *LT* lesser tubercle, *SSP* supraspinatus muscle, *SSC* subscapularis muscle, *LHB* long head of the biceps brachii muscle.

57.5% of all examined shoulder girdles showed at least a partial rupture of the CHL. (n = 23, 15 female, 8 male, 9 left, 14 right). Out of these, isolated lesions of the posterolateral band of the CHL occurred in almost all shoulders (95.7%, n = 22) (see Fig. 4), none were isolated lesions of the anteromedial band. Combined lesions of both bands and thus total ruptures of the CHL were found in 4.3% (n = 1) of all cases (see Fig. 5). The analysis of the expanse of CHL ruptures showed a mean value of 113.7 mm^2^ ± 95.1 mm^2^. A significant difference (*p* = 0.031) in rupture area of the CHL could be found between left and right shoulders (mean value left: 61 mm^2^ ± 68.6 mm; right: 150.6 mm^2^ ± 109 mm^2^). No statistically significant difference could be shown between sex (*p* = 0.277). Demographic measurements of the CHL may be found in Table 1 (see also Fig. 6 for exemplary evaluation of the osseous attachment areas).

**Table 1:** Morphology of the CHL.

	All	Women	Men	Right	Left
Coracoid insertion area (mm^2^)^a^	45.2 ± 16.2	40.2 ± 12.0	50.5 ± 18.6	47.7 ± 18.1	42.4 ± 12.3
Humeral insertion area (mm^2^**)**	58.9 ± 21.1	55.2 ± 22.3	62.8 ± 18.3	55.4 ± 17.4	63 ± 24.7
Lesser tubercle (mm^2^)	17.3 ± 9.1	15.6 ± 7.2	19 ± 10.6	17.9 ± 6.1	16.6 ± 12
Greater tubercle (mm^2^)^b^	49.2 ± 17.3	48.4 ± 17.8	50 ± 17.2	44.7 ± 12	54.3 ± 21
Width (mm)^c^	12.9 ± 5.3	11.3 ± 5.3	14.6 ± 4.8	13 ± 5.2	12.7 ± 5.5
Length (mm)^d^	48.1 ± 7.4	45.5 ± 7.1	14.6 ± 4.8	47.8 ± 8	48.5 ± 6.8

^a^Significant difference between females and males (*p* = 0.045).

^b^Strong correlation between total insertion area and area on greater tubercle r = 0.940, *p* < 0.001.

^c^Significant difference between females and males (*p* = 0.049).

^d^Significant difference between females and males (*p* = 0.17).

A relation between the presence of rotator cuff tears and ruptures of the CHL was object of investigation. Referring to this, a highly significant correlation (*p* < 0.001, φ = 0.529) between the presence of rotator cuff tears and ruptures of the CHL was found. Of the 23 cases of at least partial rupture of the CHL, an additional SSC rupture was found in 11 cases (47.8%). No isolated SSC rupture without CHL rupture was found. Results show a significant correlation between lesions of the posterolateral band and associated ruptures of the SSP (*p* = 0.001, φ = 0.529), but not for SSC lesions (*p* = 0.248) or LHB ruptures (*p* = 0.499). Lesions of the anteromedial band of the CHL and lesions of the whole CHL did not correlate with lesions of the SSC (*p* = 0.075), of the SSP (*p* = 0.275), but were correlated with lesions of the LHB (*p* = 0.050, φ = 0.698).

Anterosuperior cartilage damage of the humeral head was observed in 20% (n = 8) cases (see Fig. 5). This cartilage damage did not correlate with damage to the CHL (*p* = 0.428) or the LHB (*p* = 0.364) but was significantly associated with lesions to the SSP (*p* = 0.003, φ = 0.532) and the SSC (*p* = 0.006, φ = 0.569).

## Discussion

Data presented in this study revealed a significant correlation between RCT and ruptures of the CHL. If a rupture to the rotator cuff was identified, the CHL was always also damaged. However, ruptured CHLs were also found in joints with macroscopically uninjured tendons. These findings suggest that the development of degeneration of the rotator cuff may not be singularly supported by the classic acromial or coracoidal impingement-syndrome-theory, but occurs from articular towards the subacromial and subcoracoidal space and thus proving the existence of an anterosuperior glenoidal impingement.

The evaluation of the dissected specimens showed an overall prevalence of 27.5% of full-thickness RCT (n = 11) which is comparable to findings of other authors^7,9^. Differences may be due dissimilarities of subject population (age, clinical history etc.) and different survey methods (MRI, ultrasound, arthroscopy, anatomical dissection etc.). A higher prevalence and greater size of tears with increasing age as reported in the literature, was also confirmed in this study^7–9^.

Apart from the number of full-thickness RCT, a prevalence of 57.5% of CHL ruptures was shown, revealing a significant connection between full-thickness RCT and lesions of the CHL (*p* = 0.001). This is supported by the early dissection results of Slatis and Aalto^16^, who reported ruptures of the CHL in addition to full thickness tears of the SSP tendon in all cases (n = 5). These ruptures focussed mainly on the anteromedial portion of the CHL, leading to a dislocation of the tendon of the LHB^16^. Particularly notable was, that in all shoulders with a RCT (n = 11) the CHL was ruptured (see Fig. 5). However, there was evidence of an injured CHL in 12 shoulder without a tear of the RC. All in all, this indicates a degeneration of the RC from articular towards the subacromial space, and thus proves an anterosuperior glenoidal impingement, which is contrary to the classic subacromial or subcoracoid impingement-syndrome-theory.

The coracoid insertion of the CHL showed a mean size of 45.2 ± 16.2 mm^2^. The total insertion area on the humerus measured 58.9 ± 21.1 mm^2^, the anteromedial band of the CHL inserted in an area of 17.3 ± 9.1 mm^2^ at lesser tubercle of the humerus, the posterolateral band in an area of 49.2 ± 17.3 mm (see Fig. 6). The total insertion area and the insertion site of the posterolateral band showed a strong positive correlation (r = 0.940, *p* < 0.001), which reflected, that the insertion at the greater tubercle makes up the main part of the total insertion area on the humerus. In their recent publication, Dekker et al.^32^ presented a detailed description of the glenohumeral ligaments, including the CHL. They reported a coracoid insertions size of the ligament of 66.5 ± 23.8 mm^2^ and a humeral insertion size of 68.3 ± 18.4 mm^2^. Also, Schwarz and Hirtler^29^ in their paper on the ectopic tendons of the pectoralis minor muscle measured the coracoid insertion area of the CHL, which was overall 56.5 ± 36.3 mm^2^, ranging from 54.9 ± 34.4 mm^2^ in their group without variation to 66.31 ± 33.8 mm^2^ in their group with variation (exemplary image of an ectopic tendon of the pectoralis minor muscle may be found as supplementary material). Although the results of this study showed generally smaller insertion sites compared to the two prior publications, this mainly reflects the interindividual variability of this anatomical structure, which is also mainly supported by the sex-difference reported.

A mean width of the CHL was 12.9 ± 5.3 mm (female: 11.3 mm ± 5.3 mm; male: 14.6 ± 4.8 mm) and a mean length of 48.1 ± 7.4 mm (female: 45.5 mm ± 7.1 mm; male: 51 mm ± 6.7 mm) was found. The mean width of the CHL is in accordance with the measurements reported by Dekker et al.^32^ (12.9 mm). In contrast to Sun et al.^33^, statistically analysis pointed out a significant difference between female and male in width (*p* = 0.05) and length (*p* = 0.017) of the CHL. Dimensions in male shoulders were significantly bigger than in female. These differences may be due to a bigger general growth of men compared to women, as has been reported for numerous other anatomical structures.

The important structure passing through the rotator interval is the tendon of the LHB. Its position in the intertubercular groove and passing through the glenoid capsule is mainly stabilized by fibers of the SSP, the SSC and the complex comprised of the CHL and the SGHL.

Which of those two ligaments play a more important role in the stabilization of the LHB is still a topic of discussion. Some state that the SGHL is the most important stabilizing structure and the CHL only contributed to the tension of the SGHL^34,35^, others describe the SGHL as not robust, with a significant smaller cross-sectional area and lesser stiffness and ultimate load compared to the CHL^32,36^. Additionally, the question arises, whether this discussion is not hampered by comparison of different evaluation methods, as most publications describing SGHL lesions are preformed arthroscopically^34,37^, whereas the recognition of a ligament complex comprised of the anteromedial band of the CHL and the SGHL was initiated by dissection^32^ and adapted in the clinical context^31,38^.

Martetschlager et al.^31^ described in their recent publication the frequency and morphology of pulley lesions subdivided into cases with lesions of the lateral pulley sling (i.e. the posterolateral band of the CHL) and with lesions of the medial pulley sling (i.e. the complex comprised of the anteromedial band of the CHL and the SGHL). Lesions of the lateral pulley sling occurred in 95%, lesions of medial pulley sling in 64% of their patients (n = 100). Those lesions were isolated medially in 5%, isolated laterally in 36% and involved both slings in 59%. In addition, they reported 40 articular-sided partial tears and 48 complete tears of the SSP as well as 28 partial and 18 complete tears of the SSC^31^. This is well reflected by the results presented above, as there was a higher number of CHL ruptures found compared to RCT as well as a higher prevalence of lesions to the posterolateral band of the CHL compared to the anteromedial band (see Fig. 4).

Based on their findings, Martetschlager et al.^31^ proposed an updated classification system for biceps pulley lesions (previously introduced by Habermeyer et al.^34^), subdividing the lesions into three subtypes: type 1—lesions to the medial pulley sling (SGHL and amCHL complex), type 2—lesions to the lateral pulley sling (plCHL) and type 3 lesions to both pulley slings (SGHL and complete CHL). A type 3 lesion is significantly correlated with a higher frequency of concomitant complete SSC tears in addition to a higher frequency of fraying or partial rupture of LHB compared to type 2 lesions^31,34,39^. Applying this classification to the results of the current study, no shoulder showed a type 1, 22 shoulders a type 2 and one shoulder a type 3 lesion (see Fig. 3). Type 3 lesions were positively correlated with lesions of the LHB (*p* = 0.050, φ = 0.698).

Literature reflects that lesions to the isolated CHL or CHL-SGHL-complex are strongly associated especially with partial articular-side RCT^13,16,40–42^. These defects cannot originate from external causes as subacromial or subcoracoidal impingement, but have to be the results of an impingement of the undersurface of the biceps pulley and the adjacent tendons against the anterosuperior rim of the glenoid fossa, most likely in a position of adduction and internal rotation of the upper extremity^43,44^. This pathophysiological approach also explains why partial SSC tears are not only always identified more proximally but also consistently affect the articular side of the SSC tendon^13,39,45^. In analogy to a posterosuperior glenoid impingement, the anterosuperior glenoid impingement leads to articular side lesions^46^ and not to bursal side tears, which are rather to be expected after subacromial^47^ or subcoracoid impingement^48^. It seems like anterosuperior impingement is especially correlated to increasing age, as already suggested by Habermeyer et al.^34^ and confirmed by this study.

The presented dissection results in this study reflect well the occurrence of anterosuperior impingement especially in the elderly population, in which RCT most often develop without specific trauma. In these, always the question arises whether shoulder function in daily life activity is achieved through conservative approaches or joint instability is reported, warranting surgical therapy. Whether CHL reconstruction may be a path to pursue, future studies will have to show. However, in anterior and multidirectional instability, the reconstruction of this part of the biceps pulley or its complete closure already has been shown^49,50^.

Some limitations must be considered when evaluating this anatomical study. Measuring tear areas in rotator cuff arthropathies and CHL ruptures only allowed a post-mortem study-design. The high age of the body donors in anatomical studies is always a point of discussion. However, the specific lesions investigated in this setting especially warrants an elderly collective, as atraumatic RTC more often occur with higher age. Another topic is the effect of cryopreservation on tissue quality. This is especially important when applying imaging techniques such as MRI in an anatomical setting. As no additional imaging was performed, tissue quality for dissection was acceptable, especially as previous studies also pointed out the disadvantage of formalin-phenol fixation in terms of evaluating ligaments and tendons^29^. Particularly the distinction of different tissue layers was not affected by cryo-preservation. In addition, utmost care was taken in performing all measurements of the CHL and the respective rupture sizes. Nonetheless one has to point out, that especially in the evaluation of two-dimensional photographs of a three-dimensional structure some measurement bias exists.

In rotator cuff degeneration and atraumatic rotator cuff tears of the elderly population, the pathomechanism of full-thickness RCT is based on repetitive anterosuperior glenoid impingement of the structures of the rotator interval and the adjacent tendons of the rotator cuff. This is especially supported by the identification of a higher frequency of CHL lesions compared to RCT reported in this study. No intact CHL was identified in shoulders with damaged rotator cuff tendons.

## Methods

In total, forty-two fresh frozen upper extremities (38 paired, 4 unpaired) were examined and dissected. The specimens originated from voluntary body donations of the Center of Anatomy and Cell Biology of the Medical University of Vienna, informed consent was obtained from all subjects prior to their death for their body to be used for scientific and teaching purposes. The mean age of the body donors was 76.4 ± 10 years, with a range of 41 to 94 years. The project was approved by the ethical committee of the Medical University of Vienna (2257/2018), the study was performed in accordance with the Declaration of Helsinki and the Medical Research Involving Human Patients Act. Inclusion criteria were adequate tissue quality and absence of signs of previous surgery. Exclusion criteria were moderate to massive arthrosis and deformities of the humeral head. After applying exclusion criteria, forty anatomical specimens (20 females and 20 males, 18 left and 22 right, 36 paired, 4 unpaired) were evaluated. Two of the initially included specimens had to be excluded due to severe arthrosis and deformity of the humeral head revealed during dissection. Of the remaining 40 specimens, 60% (n = 24) of the glenohumeral joints showed mild signs of osteoarthrosis and one specimen (2.5%) a calcific tendinitis, which did not influence the dissection results. The remaining fifteen shoulders (37.5%) showed no macroscopic evidence of comorbidities. The Bateman-Classification^51^ was used to classify full-thickness rotator cuff tears (Group 1: < 1 cm, Group 2: 1–3 cm, Groupe 3: 3–5 cm, Group 4: > 5 cm).

### Dissection and evaluation

**Figure 7:**
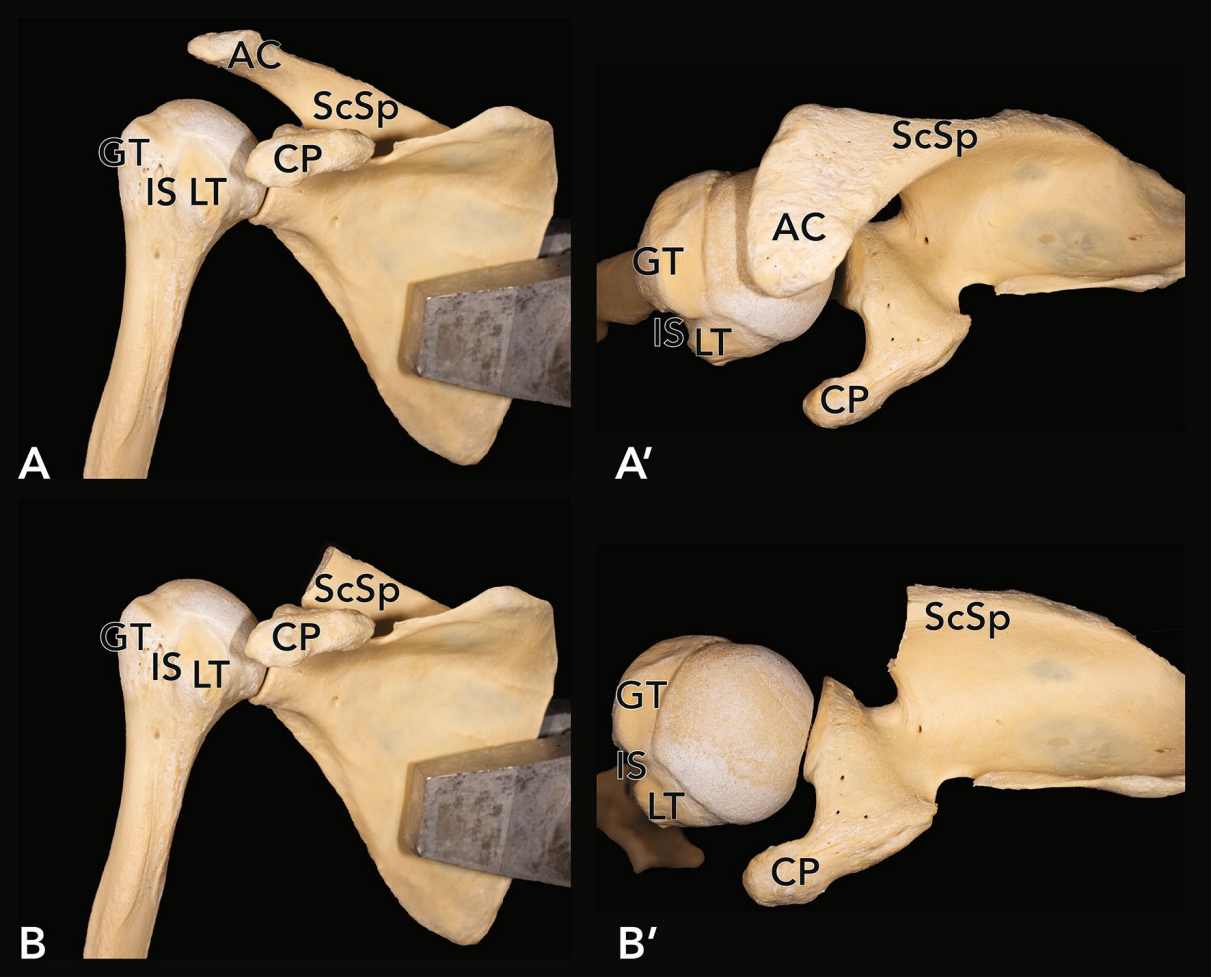
Exemplary view of the osseous morphology before (**A**, **A′**) and after (**B**, **B′**) removal of the acromion of the scapula in a right shoulder. View from anterior (**A**, **B**) and from superior (**A′**, **B′**). *AC* acromion, *CP* coracoid process, *ScSp* scapular spine, *GT* greater tubercle, *LT* lesser tubercle, *IS* intertubercular sulcus.

The specimens were mounted in a custom-made vice as commonly used in anatomical arthroscopic procedures. Soft tissue was removed to expose the rotator cuff and the CHL. Parts of the fornix humeri, the acromion (see Fig. 7) and the coracoacromial ligament in particular, were extracted by a cut between the acromion and the spine of the scapula. The muscles of the rotator cuff were detached from their origins, separated carefully from the capsule of the glenohumeral joint and mobilized, to allow full view of the capsule and the CHL. The anterior and posterior border of the CHL and its parts were identified, and the adjacent capsule was removed. The proximal and distal osseous attachments of the CHL were marked.

Every step of the dissection process was documented photographically in a standardized fashion. Each pathological finding was additionally documented separately. Direct measurements of the width and length of the CHL were performed using a sliding caliper with an accuracy of 0.01 mm. For area measurements, specific plane parallel photographs were taken in scale of the identified RCTs (rupture area of the rotator cuff and of the CHL) and of the humeral and coracoid insertion areas of the CHL. These measurements were performed using ImageJ^®^ (https://imagej.nih.gov/ijgov/ij). Comorbidities like arthrosis, tendinopathies and lesions of the long biceps tendon were documented during the dissection process.

### Statistics

Statistical analysis was performed using SPSS Statistics (IBM Corp. Released 2018. IBM SPSS Statistics for Windows, Version 25.0. Armonk, NY: IBM Corp). For all metric data mean, standard deviation and range were documented. Normal distribution of data was evaluated by visualization in boxplots and by the Shapiro–Wilk test. Fisher’s exact test was used to determine whether there is a statistically significant difference between the expected and observed frequencies in a contingency table (n < 20). Differences were assessed using the Student’s t-test. For the correlation between areas (origin and insertion of the CHL, rupture areas of the CHL and rotator cuff tears) a Pearson-Correlation was performed as data were distributed normally. The correlation coefficient was interpreted as follows: ± 0.7–1 strong, ± 0.5–0.7 moderate, ± 0.3–0.5 low, <  ± 0.3 weak correlation. A *p* value < 0.05 was considered as statistically significant.

### Ethical approval

The study was approved by the ethical committee of the Medical University of Vienna (EK Nr. 2257/2018).

## Supplementary Information

Supplementary Information 1.

Supplementary Information 2.
