# Supplementary figures and images for: Association of damage to the coracohumeral ligament with anterosuperior rotator cuff degeneration revealed by anatomical dissection
Source: Sci Rep. 2022 Mar 10;12:4238. doi: 10.1038/s41598-022-08070-x (PMC8913693; doi:10.1038/s41598-022-08070-x)

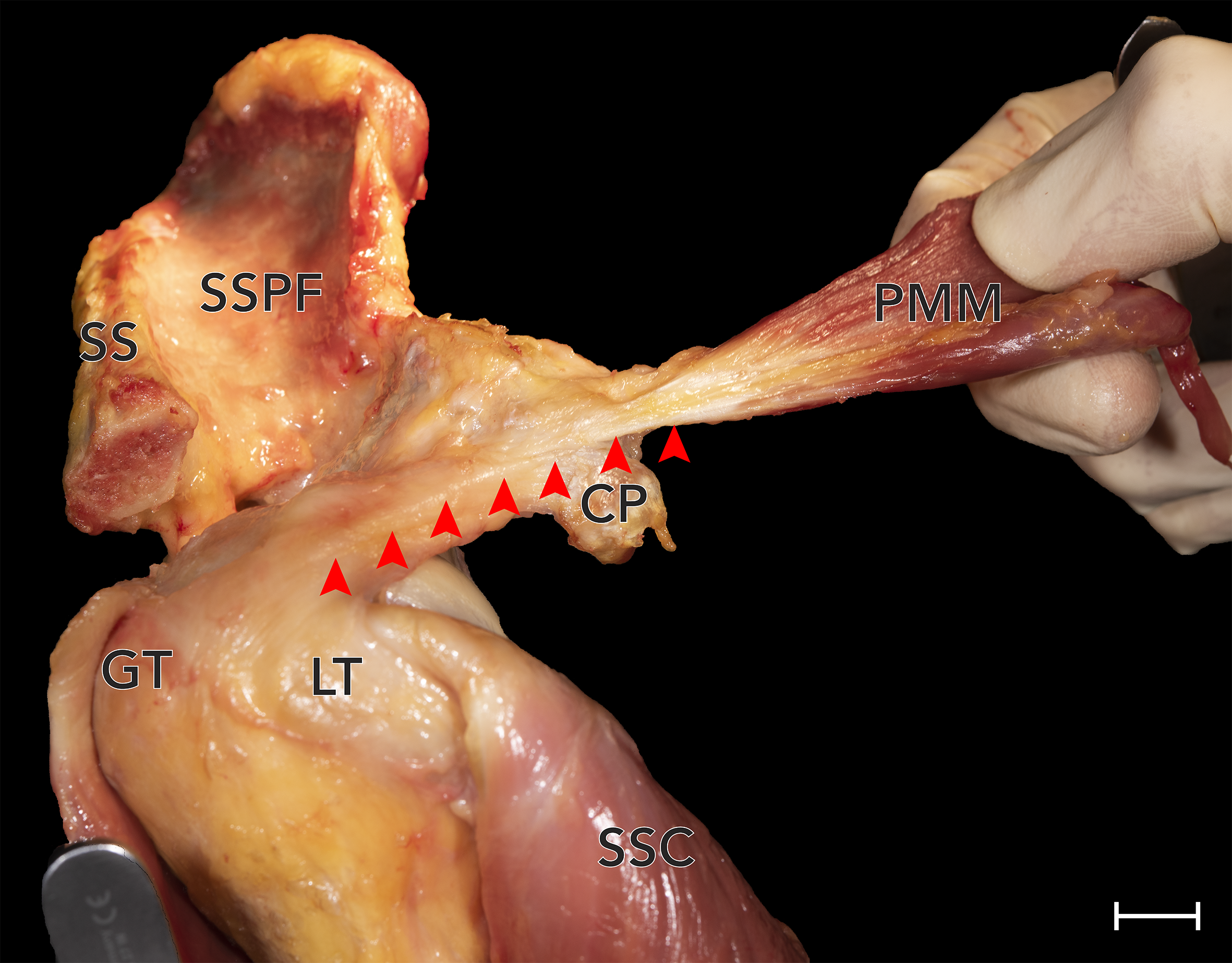

Supplement: Supplementary file 1 — Supplementary Information 1. [file 41598_2022_8070_MOESM1_ESM.tif]
