# Supplementary material for: Association of damage to the coracohumeral ligament with anterosuperior rotator cuff degeneration revealed by anatomical dissection
Source: Sci Rep. 2022 Mar 10;12:4238. doi: 10.1038/s41598-022-08070-x (PMC8913693; doi:10.1038/s41598-022-08070-x)
Supplement: Supplementary file 2 — Supplementary Information 2. [file 41598_2022_8070_MOESM2_ESM.docx]

**Supplementary Figure 1** Case of an ectopic insertion of the pectoralis minor muscle in our study population. Continuation of the fibers of the tendon of the pectoralis minor muscle into the CHL is clearly visible (read arrowheads). CP – coracoid process, SS – scapular spine, SSPF – supraspinous fossa, GT – greater tubercle, LT – lesser tubercle, SSP – supraspinatus muscle, SSC – subscapularis muscle.
